# Aging in females is associated with changes in respiratory modulation of sympathetic nerve activity and blood pressure

**DOI:** 10.1152/ajpheart.00226.2023

**Published:** 2023-09-01

**Authors:** Zoe H. Adams, Hazel C. Blythe, Nisha Charkoudian, Timothy B. Curry, Michael J. Joyner, Adrian H. Kendrick, Angus K. Nightingale, Ana P. Abdala Sheikh, Emma C. Hart

**Affiliations:** ^1^School of Physiology, Pharmacology and Neuroscience, University of Bristol, Bristol, United Kingdom; ^2^Translational Health Sciences, University of Bristol, Bristol, United Kingdom; ^3^United States Army Research Institute of Environmental Medicine, Natick, Massachusetts, United States; ^4^Department of Anaesthesiology, Mayo Clinic, Rochester, Minnesota, United States; ^5^Department of Respiratory Medicine, University Hospitals Bristol and Weston NHS Foundation Trust, Bristol, United Kingdom; ^6^Bristol Heart Institute, University Hospitals Bristol and Weston NHS Foundation Trust, Bristol, United Kingdom

**Keywords:** blood pressure, menopause, respiration, sex, sympathetic

## Abstract

Sympathetic nerve activity (SNA) is tightly coupled with the respiratory cycle. In healthy human males, respiratory modulation of SNA does not change with age. However, it is unclear how this modulation is affected by age in females. We investigated whether respiratory sympathetic modulation is altered in healthy postmenopausal (PMF) versus premenopausal female (YF), and younger male (YM) adults, and determined its relationship to resting blood pressure. Muscle SNA (MSNA; microneurography), respiration (transducer belt), ECG, and continuous blood pressure were measured in 12 YF, 13 PMF, and 12 YM healthy volunteers. Respiratory modulation of MSNA was quantified during two phases of the respiratory cycle: mid-late expiration and inspiration/postinspiration. All groups showed respiratory modulation of MSNA (*P* < 0.0005). There was an interaction between the respiratory phase and group for MSNA [bursts/100 heartbeats (HB) (*P* = 0.004) and bursts/min (*P* = 0.029)], with smaller reductions in MSNA during inspiration observed in PMF versus the other groups. Respiratory modulation of blood pressure was also reduced in PMF versus YF (6 [2] vs. 12 [9] mmHg, *P* = 0.008) and YM (13 [13] mmHg, *P* = 0.001, median [interquartile range]). The magnitude of respiratory sympathetic modulation was related to resting blood pressure in PMF only, such that individuals with less modulation had greater resting blood pressure. The data indicate that aging in postmenopausal females is associated with less inspiratory inhibition of MSNA. This correlated with a higher resting blood pressure in PMF only. Thus, the reduced modulation of MSNA could contribute to the age-related rise in blood pressure that occurs in females.

**NEW & NOTEWORTHY** The current study demonstrates that respiratory modulation of sympathetic nerve activity (SNA) is reduced in healthy postmenopausal (PMF) versus premenopausal females (YF). Furthermore, respiratory sympathetic modulation was negatively related to resting blood pressure in postmenopausal females, such that blood pressure was greater in individual with less modulation. Reduced respiratory sympathetic modulation may have implications for the autonomic control of blood pressure in aging postmenopausal females, by contributing to age-related sympathetic activation and reducing acute, respiratory-linked blood pressure variation.

## INTRODUCTION

Sympathetic vasomotor activity is modulated by phase of the respiratory cycle in a species-dependent manner (1). In humans, sympathetic nerve activity (SNA) directed to the smooth muscle in the vasculature (muscle SNA; MSNA) is greatest toward the end of expiration and is inhibited at and shortly after the inspiratory peak (2, 3). Loss or blunting of respiratory modulation of SNA is associated with higher overall MSNA; for example, in patients with chronic heart failure, MSNA was highest in those with the least inspiratory inhibition of MSNA (4). Interestingly, in male adults, healthy aging does not appear to affect respiratory sympathetic modulation, given that MSNA was similarly modulated in older and younger healthy male participants (5). However, there is a dearth of information and studies regarding what happens to respiratory control of SNA and blood pressure in aging females who have undergone menopausal transition. Indeed, whether respiratory sympathetic modulation is similarly maintained in postmenopausal females (PMF) is unknown.

Female aging (including menopausal transition) is associated with changes to autonomic and respiratory regulation that have consequences for the health of older females. Not only do levels of SNA and blood pressure rise with age, but blood pressure regulation is more heavily dependent on SNA in older (postmenopausal) versus younger (premenopausal) females (YF) (6, 7). Estrogen is known to inhibit sympathetic outflow (8), thus the decline in estrogen at menopause could drive sympathetic activation. Therefore, increased SNA and tighter coupling between SNA and blood pressure may expose older females to increased hypertension risk, which can be in excess of that in age-matched males (9, 10). In both males and females, respiratory function (forced vital capacity, forced expiratory volume in 1 s, peak expiratory flow rate) declines with age (11), and inspiratory muscle function, as measured by maximal inspiratory pressures, also appears reduced in older versus younger adults (12, 13). Importantly, menopause appears to affect age-related changes in respiratory function. Age-adjusted lung function was lower (14) and annual decline in lung function was greater in postmenopausal versus younger, premenopausal females (15). Furthermore, across several age groups earlier menopausal transition was associated with poorer lung function (14) and lung function in postmenopausal females correlates positively with reproductive lifespan (16). Taken together, these reports indicate that both sympathetic and respiratory function is influenced by aging and menopausal transition. As such, respiratory sympathetic modulation may be impacted by age specifically in females and, as such, contributes to the age-related increase in SNA in females. Therefore, we aimed to determine whether respiratory sympathetic modulation (and respiratory-linked oscillations in blood pressure; Traube–Hering waves) is impacted by aging in females by comparing healthy postmenopausal females to younger, premenopausal females. We hypothesized that respiratory sympathetic modulation would differ between premenopausal and postmenopausal females. We additionally examined whether any change in the respiratory modulation of SNA with age was related to the overall resting level of SNA and blood pressure. We hypothesized that there would be a negative relationship between the degree of respiratory sympathetic modulation and resting level of SNA. Finally, given that respiratory sympathetic modulation has not yet been compared between males and females, we aimed to determine whether any sex difference in respiratory sympathetic modulation exists in young healthy adults. Existing work indicates that baroreflex modulation of SNA firing is similar among young males and females (17, 18), therefore we hypothesized that respiratory modulation of SNA would also be similar in the younger male (YM) and female participants.

## METHODS

### Ethical Approval

The study in which data were collected (19) received ethical approval from the Mayo Clinic Institutional Review Board and conformed to the standards of the Declaration of Helsinki at the time of data collection. Participants gave written informed consent to participate.

### Participants

Data from 12 premenopausal female, 12 younger male, and 13 postmenopausal female participants were analyzed retrospectively. Participants were recruited as male or female according to self-report. Participants were healthy and reported no current or history of cardiovascular or respiratory disease. Regular medication other than hormonal contraception was not used by any participant. Premenopausal females took part during the early follicular phase of the menstrual cycle, or during the low-hormone phase of hormonal contraception use. Postmenopausal females reported amenorrhea for at least 1 yr and did not use hormone replacement therapy.

### Experimental Procedures

MSNA was recorded by microneurography, using established protocols (20). A tungsten microelectrode was inserted into the common peroneal nerve and manipulated until a suitable site was identified. The signal was confirmed as MSNA by the presence of narrow pulse-synchronous bursts and the absence of response to tactile or startle stimuli (20). The microneurographic signal was amplified 80,000 times, bandpass-filtered (0.7–2 kHz), rectified, and integrated (0.1-s time constant) (Nerve Traffic Analyzer, University Iowa Bioengineering). ECG and respiration were recorded by three-lead ECG and force transducer placed around the abdomen, respectively. Continuous blood pressure was measured by a pressure transducer inserted into the brachial artery under 2% lidocaine. MSNA, ECG, respiration, and blood pressure were recorded over a 5- to 10-min period of quiet rest while the participant lay semisupine.

### Data Analysis

Data were analyzed blind to participant group. Bursts of MSNA were identified from the integrated neurogram using a custom script (Spike2, v.8.13, Cambridge Electronic Design, Cambridge, UK). Heart rate was calculated beat to beat between consecutive R waves, whereas systolic, diastolic, pulse pressure, and mean arterial pressure were calculated at every blood pressure waveform. Across the entire analysis window, MSNA was expressed as bursts/100 heartbeats (HB), bursts/min, and total burst area/s. MSNA area (modulus of the integrated neurogram, normalized to the tallest burst) was sampled during inspiratory/expiratory intervals (see *Respiratory Sympathetic Modulation Analysis*), thus total area/s was calculated as the sum of burst area across all of these intervals, divided by the duration of the analysis window. Heart rate and blood pressure variables were averaged across the analysis window. Respiratory trace amplitude was calculated as the peak-to-peak amplitude between adjacent end-expiratory markers, normalized to the largest breath within each individual and averaged across the entire analysis window. Respiratory rate was calculated across the entire analysis window. Spontaneous sympathetic baroreflex sensitivity (sBRS) of burst occurrence and area were determined by an established method (21) using a custom script (Spike2, Cambridge Electronic Design, Cambridge, UK). Briefly, for each diastolic blood pressure, it was determined whether a burst occurred in the following cardiac cycle (burst occurrence), and the modulus of the integrated neurogram (normalized to the tallest burst and shifted backward by mean burst latency) at ±0.4 s around the R wave of the following cardiac cycle was sampled (burst area). Probability of burst occurrence and mean burst area were then determined for 1-mmHg bins of diastolic blood pressure. The slope of the weighted linear regression between diastolic blood pressure and either probability of burst occurrence or mean burst area was taken as the sBRS for each individual.

### Respiratory Sympathetic Modulation Analysis

**Figure 1.**
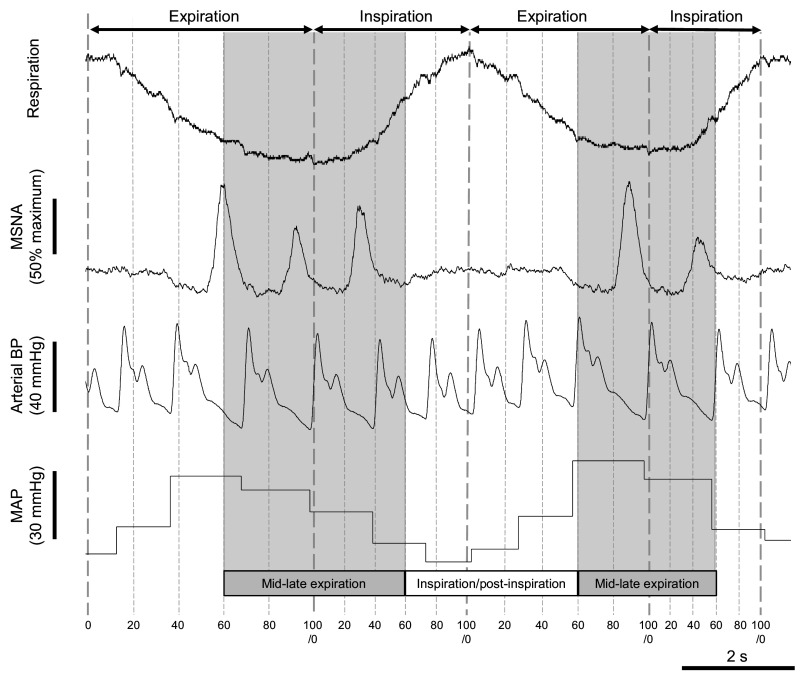
Example respiratory trace showing the mid-late expiration and inspiration/postinspiration analysis phases. BP, blood pressure; MAP, mean arterial pressure; MSNA, muscle sympathetic nerve activity. The MSNA neurogram was normalized to the tallest burst and shifted backward by mean burst latency. MAP was quantified as the modulus of each arterial blood pressure waveform and changes in MAP across the respiratory cycle represent respiratory modulation of blood pressure (Traube–Hering waves). Percentage of respiratory phase is displayed along the bottom of the figure. Data are from one premenopausal female participant.

Analysis of respiratory sympathetic modulation was conducted using a custom script (Spike2, Cambridge Electronic Design, Cambridge, UK). Inspiratory and expiratory periods of the respiratory cycle were considered separately, with inspiration defined as trough to peak of the respiratory trace and expiration defined as peak to trough. Each respiratory period (inspiratory or expiratory) was divided into five equal intervals over which MSNA data were sampled. Before extraction of data, the MSNA neurogram was shifted backward by individual mean burst latency (the time delay between a MSNA burst and the R wave inhibiting further sympathetic firing during a particular cardiac cycle). MSNA burst event and R wave data were extracted using a publicly available script [RasterDump.s2s (original version), Spike2, Cambridge Electronic Design, Cambridge, UK], whereas MSNA burst area was measured using the custom script as the modulus of the integrated shifted neurogram, normalized to the tallest burst in the analysis window. Respiratory phases aligned with artifacts in the neurogram were removed. MSNA burst incidence, burst frequency, and burst area normalized for interval duration (burst area/s) were then calculated across two respiratory phases, as previously described (5): inspiration/postinspiration, represented by intervals 60–100% of inspiration and 0–60% of expiration; and mid-late expiration, represented by intervals 60–100% of expiration and 0–60% of inspiration (Fig. 1).

### Analysis of Respiratory Sinus Arrhythmia and Traube–Hering Waves

Respiratory modulation of heart rate (respiratory sinus arrhythmia) was quantified for each respiratory cycle as the maximum absolute change in beat-to-beat heart rate occurring between adjacent end-expiration points. Respiratory modulation of blood pressure (Traube–Hering waves, Fig. 1) was measured as the maximum absolute change in beat-to-beat mean arterial blood pressure between one end-expiration point and the next end-expiration point plus 1 s; mean arterial blood pressure was calculated as the modulus of each blood pressure waveform between adjacent diastolic blood pressures (5). The measures of respiratory sinus arrhythmia and Traube–Hering wave amplitude were averaged across the entire analysis window.

### Relationships between Respiratory-Modulated and Resting Hemodynamic Variables

To further interrogate the relationship between respiratory-modulated MSNA and blood pressure, respiratory-modulated MSNA was correlated with Traube–Hering wave amplitude of the same respiratory cycle, the following cycle (+1 lag), and the previous cycle (−1 lag) (5). For these correlations, MSNA was quantified as the modulus of the original, unshifted integrated neurogram for 1.5 times the duration of inspiration, from the start of inspiration, as in Supplemental Fig. S1 (all Supplemental material is available at https://doi.org/10.6084/m9.figshare.22673056) (5). Furthermore, to investigate whether respiratory sympathetic modulation was related to resting hemodynamic variables, the percent change in MSNA burst incidence between the mid-late expiration and inspiration/postinspiration respiratory phases was correlated with average resting systolic, diastolic, and mean arterial blood pressure across the analysis window, as well as resting level of MSNA burst incidence.

### Statistical Analysis

Statistical analysis was conducted in SPSS statistics (v.24, IBM, New York, NY) and data were graphed in GraphPad Prism (v.9.4.0, GraphPad Software, San Diego, CA). Group differences in respiratory sympathetic modulation were tested by mixed-model ANOVA, whereas group differences in participant characteristics, hemodynamic variables, and correlation coefficients were assessed by one-way ANOVA or Kruskal–Wallis test. Correlations between respiratory and hemodynamic variables were determined by Spearman’s rank correlation or Pearson’s correlation. Either χ^2^ or Fisher exact test was used to determine whether there were group differences in the proportion of participants with significant correlations between variables. Data are expressed as means ± SD or medians [interquartile ranges] throughout and *P* < 0.05 was considered significant.

## RESULTS

### Participant Characteristics

Participants’ characteristics are shown in Table 1. Premenopausal female and younger male participants were matched for age and were younger than the postmenopausal female group. Body mass index was similar across all groups. Data on hormonal contraception use were available in 11 of 12 premenopausal female participants (Table 1); six participants used oral contraceptives, one used a vaginal ring, and four reported no hormonal contraception use.

**Table 1.** Participant characteristics in YF, YM, and PMF

	YF	YM	PMF	Test Statistic	*P* Value
Age, yr	25 [5]*	26 [7]*	58 [8]	χ^2^(2) = 24.737	***<0.0005***
Height, m	1.67 [0.10]†	1.76 [0.07]	1.67 [0.10]†	χ^2^(2) = 15.329	***<0.0005***
Weight, kg	64 [11]†	78 [12]	67 [11]†	χ^2^(2) = 1.735	***0.005***
BMI, kg/m^2^	23.8 ± 1.1	24.7 ± 2.2	24.2 ± 2.5	*F*(2, 29) = 0.721	0.495
Hormonal contraception use in YF (of *n* = 12)	None: 4 of 12 OC: 6 of 12 Vaginal ring: 1 of 12 N/A: 1 of 12				

Values are represented as means ± SD or medians [interquartile ranges]. BMI, body mass index; OC, oral contraception; N/A, not available; χ, Kruskal–Wallis test statistic; *F*, ANOVA test statistic. Multiple comparisons for height and weight compared mean ranks. Height, weight, and BMI data available for 11 of 12 premenopausal females (YF), 12 of 12 younger males (YM), and 9 of 13 postmenopausal females (PMF). Group differences tested by univariate ANOVA or Kruskal–Wallis test.

* *P* < 0.05 vs. PMF; †*P* < 0.05 vs. YM (exact *P* values in text).

Resting MSNA was greater in postmenopausal females versus both premenopausal females (*P* < 0.0005 and *P* = 0.001; bursts/100 HB and bursts/min, respectively) and younger males (*P* < 0.0005 and *P* < 0.0005; Table 2) but did not differ between the younger groups (*P* = 1.0 and *P* = 1.0). Similarly, MSNA burst area (Table 2) was greater in postmenopausal females versus the younger groups (*P* = 0.002 vs. premenopausal females; *P* = 0.005 vs. younger males) but did not differ between the younger groups (*P* = 1.0). There were no group differences in resting heart rate (*P* = 0.247) or breathing rate (*P* = 0.818). Systolic blood pressure was greater in postmenopausal females versus younger males (*P* = 0.003) but not versus premenopausal females (*P* = 0.119). Pulse pressure in younger males was smaller versus both postmenopausal females (*P* = 0.002) and premenopausal females (*P* = 0.023). There were no group differences in diastolic (*P* = 0.378) or mean arterial blood pressure (*P* = 0.065). Spontaneous sBRS (measured by bursts/100 heartbeats) differed between the groups (*P* = 0.015), with spontaneous sBRS lower in the postmenopausal versus the younger male group (*P* = 0.012) but not versus the younger female group (*P* = 0.738). Spontaneous sBRS (measured by burst area) was similar between the groups (*P* = 0.638).

**Table 2.** Resting hemodynamic variables in YF, YM, and PMF

	YF	YM	PMF	Test Statistic	*P* Value
MSNA, bursts/100 HB	53 [13]*	51 [11]*	79 [17]	χ^2^(2) = 20.131	***<0.0005***
MSNA, bursts/min	33 [10]*	29 [6]*	52 [13]	χ^2^(2) = 21.090	***<0.0005***
MSNA, total area/s, %	8.6 [3.4]*	8.4 [1.6]*	10.9 [3.3]	χ^2^(2) = 14.922	***0.0006***
Heart rate, beats/min	62 ± 10	58 ± 9	64 ± 7	*F*(2, 34) = 1.458	0.247
SBP, mmHg	136 [17]	125 [8]*	146 [21]	χ^2^(2) = 10.866	***0.004***
DBP, mmHg	78 [13]	69 [9]	72 [13]	χ^2^(2) = 1.945	0.378
PP, mmHg	59 ± 7†	52 ± 5*	71 ± 16	*F*(2,21.1) = 10.545	***0.001***
MAP, mmHg	98 [12]	88 [8]	97 [14]	χ^2^(2) = 5.474	0.065
sBRS occurrence, % bursts/mmHg	−5.3 [1.6]	−7.1 [4.3]*	−3.4 [3.7]	χ^2^(2) = 8.423	***0.015***
sBRS area, %/mmHg	−0.51 ± 0.29	−0.62 ± 0.28	−0.56 ± 0.25	*F*(2, 34) = 0.456	0.638
Breathing rate, breaths/min	14 [2]	13 [6]	13 [5]	χ^2^(2) = 0.401	0.818
Normalized respiratory trace amplitude, %	46 ± 15	55 ± 17	50 ± 19	*F*(2, 34) = 0.829	0.445
Respiratory cycle duration, s	4.1 [0.6]	4.5 [2.1]	4.6 [1.7]	χ^2^(2) = 0.707	0.702

Values are represented as means ± SD or medians [interquartile ranges]. YF, premenopausal females; HB, heartbeats; sBRS, sympathetic baroreflex sensitivity; χ, Kruskal–Wallis test statistic; *F*, ANOVA test statistic. Group differences tested by univariate ANOVA or Kruskal–Wallis test. Welch’s correction was applied to pulse pressure (PP) data.

* *P* < 0.05 vs. postmenopausal females (PMF); †*P* < 0.05 vs. younger males (YM) (exact *P* values in text). Multiple comparisons for muscle sympathetic nerve activity (MSNA), systolic blood pressure (SBP), diastolic blood pressure (DBP), mean arterial pressure (MAP), breathing rate compared mean ranks.

### Respiratory Sympathetic Modulation of Burst Occurrence

**Figure 2.**
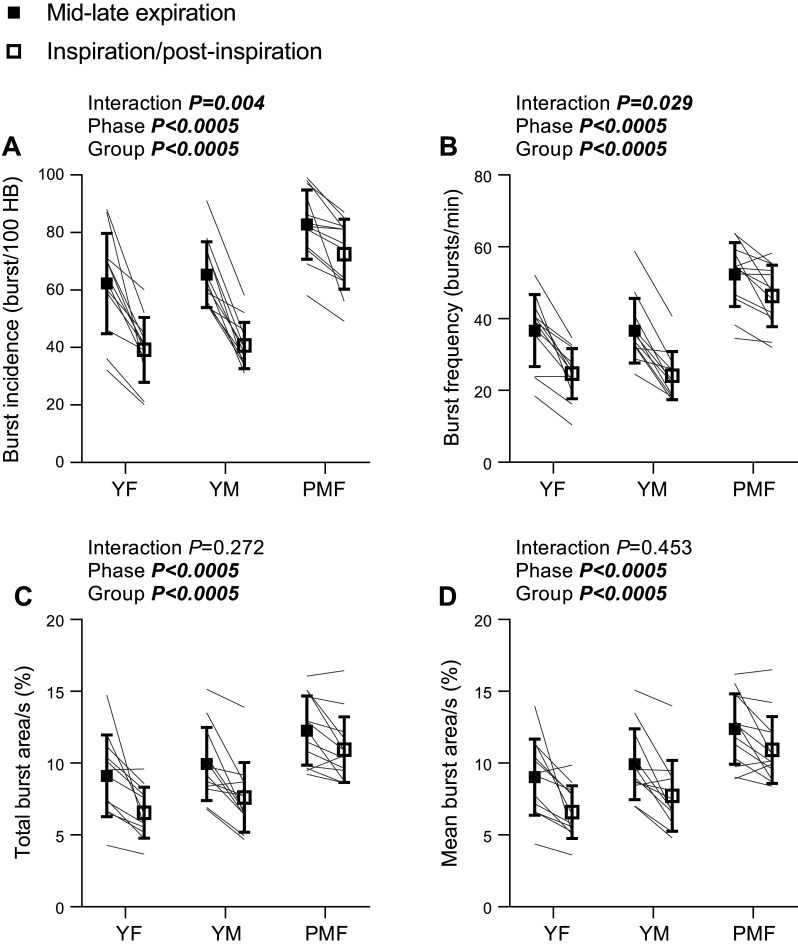
Respiratory sympathetic modulation of burst incidence (*A*), burst frequency (*B*), total burst area/s (*C*), and mean burst area/s (*D*). HB, heartbeats; PMF, postmenopausal females (*n* = 13); YF, premenopausal females (*n* = 12); YM, younger males (*n* = 12). Data are represented as means ± SD. Two-way mixed-model ANOVA.

Respiratory modulation of MSNA burst incidence and frequency between mid-late expiration and inspiration/postinspiration is shown in Fig. 2, *A* and *B*. There was a significant interaction between respiratory phase and group for both burst incidence [*F*(2,34) = 6.383, *P* = 0.004, partial η^2^ = 0.273] and burst frequency [*F*(2,34) = 3.931, *P* = 0.029, partial η^2^ = 0.188]. MSNA was reduced during the inspiratory versus the expiratory phase in all groups (Supplemental Table S1), indicating that respiratory sympathetic modulation occurred in every group. However, the change in MSNA between the respiratory phases was smaller in the postmenopausal female versus the younger groups, meaning that there was a smaller inhibition of MSNA during inspiration in postmenopausal females. Absolute and percent change in bursts/100 HB from mid-late expiration to inspiration/postinspiration was different between the groups [absolute change χ^2^(2) = 12.285, *P* = 0.002; percent change χ^2^(2) = 15.875, *P* < 0.0005] and was smaller in postmenopausal females versus both premenopausal females [*P* = 0.019 and 0.003, for absolute and percent change, respectively] and younger males [*P* = 0.004 and 0.001; Supplemental Table S2]. Similarly, percent change in burst/min differed between the groups [*F*(2,34) = 10.893, *P* < 0.0005] and was smaller in postmenopausal females versus both premenopausal females (*P* = 0.001) and younger males (*P* = 0.001; Supplemental Table S2). Absolute change in burst frequency was also different between the groups [χ^2^(2) = 7.161, *P* = 0.028] and was smaller in postmenopausal females versus younger males (*P* = 0.038) but not versus premenopausal females (*P* = 0.121; Supplemental Table S2). Neither absolute, nor percent change in bursts/100 HB (*P* = 1.0 for both) or bursts/min (*P* = 1.0 and 0.951, respectively) differed between the younger groups. When groups were compared at each respiratory phase, MSNA was greater in the postmenopausal female group versus the younger groups during both mid-late expiration and inspiration/postinspiration (Supplemental Table S1). There was no difference between MSNA in the younger groups at either respiratory phase (Supplemental Table S1).

### Respiratory Modulation of Burst Area

Respiratory modulation of total MSNA burst area/s and mean MSNA burst area/s is shown in Fig. 2, *C* and *D*. There was no interaction between respiratory phase and group for either total burst area/s [*F*(2,34) = 1.352, *P* = 0.272, partial η^2^ = 0.074] or mean burst area/s [*F*(2,34) = 0.810, *P* = 0.453, partial η^2^ = 0.045]. For both measures of burst area, there was a significant main effect of respiratory phase [total burst area/s *F*(1,34) = 39.226, *P* < 0.0005, partial η^2^ = 0.536; mean burst area/s *F*(1,34) = 37.057, *P* < 0.0005, partial η^2^ = 0.522], with reduced burst area during the inspiratory versus expiratory phase (total burst area/s 8.4 ± 2.9 vs. 10.5 ± 2.9%; mean burst area/s 8.4 ± 2.2 vs. 10.4 ± 2.5%). Furthermore, there was a significant main effect of group [total burst area/s *F*(2,34) = 10.199, *P* < 0.0005, partial η^2^ = 0.375; mean burst area/s *F*(2,34) = 10.723, *P* < 0.0005, partial η^2^ = 0.387]. Both total burst area/s and mean burst area/s were greater in the postmenopausal female versus the premenopausal female and younger male groups [total burst area/s 11.6 ± 2.2 vs. 7.8 ± 2.2% (*P* < 0.0005) and vs. 8.8 ± 2.2% (*P* = 0.0008); mean burst area/s 11.6 ± 2.2 vs. 7.8 ± 2.2% (*P* < 0.0005) and vs. 8.8 ± 2.2% (*P* = 0.008)]. Absolute change in total burst area/s between respiratory phases, and absolute and percent change in mean burst area/s between respiratory phases did not differ by group (Supplemental Table S2). There was a group difference in percent change in total burst area/s between respiratory phases [χ^2^(2) = 6.684, *P* = 0.0935], although there were no significant pairwise comparisons once corrected for multiple comparisons (Supplemental Table S2). In summary, the effect of respiratory phase on MSNA burst area was broadly similar in all groups.

### Respiratory Sinus Arrhythmia and Traube–Hering Waves

**Figure 3.**
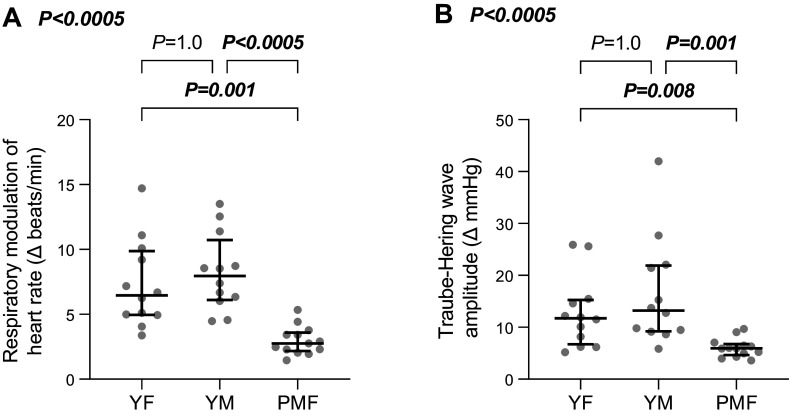
Group differences in respiratory sinus arrhythmia (*A*) and Traube–Hering wave amplitude (*B*; Δ, change values across the respiratory cycle). PMF, postmenopausal females (*n* = 13); YF, premenopausal females (*n* = 12); YM, younger males (*n* = 12). Data are medians ± interquartile ranges. Kruskal–Wallis test. Pairwise comparisons for both variables compared mean ranks.

We also examined whether respiratory modulation of heart rate (respiratory sinus arrhythmia) and blood pressure (Traube–Hering waves) were different in postmenopausal females versus premenopausal females and younger males. Group differences in respiratory sinus arrhythmia and Traube–Hering wave amplitude are shown in Fig. 3. The maximal change in heart rate between adjacent end-expiration points of the respiratory cycle (a measure of respiratory sinus arrhythmia, Fig. 3*A*) differed between groups [χ^2^(2) = 21.45, *P* < 0.0005] and was smaller in the postmenopausal female group versus both the premenopausal female (3 [1] vs. 6 [5] beats/min, *P* = 0.001) and younger male groups (8 [5] beats/min, *P* < 0.0005). There was no difference between young males and females (*P* = 1.0). These data suggest that the respiratory-linked oscillations in heart rate were smaller in postmenopausal females versus the younger groups.

Average Traube–Hering wave amplitude (Fig. 3*B*) differed between the groups (χ^2^(2) = 15.80, *P* < 0.0005), with smaller Traube–Hering waves observed in the postmenopausal female group versus both premenopausal females (6 [2] vs. 12 [9] mmHg, *P* = 0.008) and younger males (13 [13] mmHg, *P* = 0.001), but no difference observed between the younger groups (*P* = 1.0). This indicates that respiratory-linked oscillations in blood pressure were smaller in the postmenopausal female group.

### Relationship between Respiratory-Modulated MSNA and Blood Pressure

To further investigate the cause of reduced respiratory-related oscillations in blood pressure (Traube–Hering waves), we assessed the relationship between these oscillations and respiratory-modulated MSNA within the same breath and across adjacent breaths (data available in all premenopausal females, 11 of 12 younger males, and 12 of 13 postmenopausal females). In each participant, Traube–Hering wave amplitude was correlated with a measure of respiratory-modulated MSNA within the same respiratory cycle. This produced an individual correlation coefficient, where a positive coefficient indicates that a greater respiratory-modulated MSNA is associated with larger amplitude Traube–Hering waves. For these correlations, respiratory-modulated MSNA was sampled as shown in Supplemental Fig. S1 and in Ref. 5. This measure of MSNA did not differ between the participant groups [1.69 [0.74], 1.56 [0.70] and 1.69 [0.74] V·s for the premenopausal female, younger male, and postmenopausal female groups, respectively; χ^2^(2) = 0.482, *P* = 0.786]. Most participants showed a positive relationship between respiratory-modulated MSNA and Traube–Hering wave amplitude (92% premenopausal females, 82% younger males, 75% postmenopausal females; Supplemental Table S3). The correlation was significant in 67% of premenopausal females, 36% of younger males, and 25% of postmenopausal females, all of which showed a positive relationship, indicating a positive correlation between the magnitude of respiratory-modulated MSNA and Traube–Hering wave amplitude. The coefficient for the correlation between respiratory-modulated MSNA and Traube–Hering wave amplitude did not differ between groups [*F*(2) = 1.864, *P* = 0.172, Supplemental Fig. S1], indicating that Traube–Hering waves were associated with respiratory-modulated MSNA similarly across the groups. As such, the smaller Traube–Hering waves observed in the postmenopausal group appear to be the result of the reduced respiratory sympathetic modulation in the group, and not a dissociation of Traube–Hering waves from respiratory-modulated MSNA. Correlations between respiratory-modulated MSNA and Traube–Hering waves of the following breath (+1 lag) or the previous breath (−1 lag) were significant in very few participants (+1 lag) and ∼25% of participants in each group (−1 lag), respectively. There were no group differences in correlation coefficient (*P* = 0.180 and *P* = 0.448 for +1 and −1 lags, respectively), or proportion of participants with significant correlations (*P* = 0.758 and *P* = 1.0). Thus, the relationship between respiratory-linked MSNA and blood pressure across adjacent respiratory cycles was similar in all groups.

### Relationship between Resting Hemodynamic Variables and Respiratory Sympathetic Modulation

Resting blood pressure and MSNA variables were correlated with the percent change in MSNA burst incidence between the mid-late expiration and inspiration/postinspiration respiratory phases. When all participants were considered, percent change in burst incidence was positively correlated with resting systolic (ρ = 0.584, *P* < 0.0005), diastolic (ρ = 0.365, *P* = 0.026), and mean arterial blood pressure (ρ = 0.521, *P* = 0.001), such that participants who had a smaller inspiratory inhibition of MSNA had a higher resting blood pressure (or vice versa; Table 3). Interestingly, when the participant groups were considered separately, these correlations were maintained in the postmenopausal female group only. When all participants were considered, percent change in burst incidence between the respiratory phases was correlated with resting MSNA burst incidence (ρ = 0.520, *P* = 0.001), such that individuals with greater resting MSNA had a smaller inspiratory inhibition of MSNA. However, this correlation was not present in any participant group when the groups were considered separately (Table 3).

**Table 3.** Correlations between hemodynamic variables and respiratory sympathetic modulation

Correlation	Correlation Coefficient	*P* Value
Resting SBP vs. %ΔMSNA burst incidence		
All participants	ρ = 0.584	***<0.0005***
YF only	*r* = 0.407	0.189
YM only	ρ = 0.140	0.665
PMF only	ρ = 0.577	***0.039***
Resting DBP vs. %ΔMSNA burst incidence		
All participants	ρ = 0.365	***0.026***
YF only	*r* = 0.348	0.268
YM only	ρ = 0.189	0.557
PMF only	ρ = 0.692	***0.009***
Resting MAP vs. %ΔMSNA burst incidence		
All participants	ρ = 0.521	***0.001***
YF only	*r* = 0.398	0.200
YM only	ρ = 0.266	0.404
PMF only	ρ = 0.714	***0.006***
Resting MSNA burst incidence vs. %ΔMSNA burst incidence		
All participants	ρ = 0.520	***0.001***
YF only	ρ = −0.182	0.572
YM only	ρ = 0.014	0.966
PMF only	ρ = 0.286	0.344

*n* = 37 for correlations with all participants: *n* = 12 premenopausal females (YF); *n* = 12 younger males (YM); *n* = 13 postmenopausal females (PMF). SBP, systolic blood pressure; DBP, diastolic blood pressure; MAP, mean arterial pressure; MSNA, muscle sympathetic nerve activity; %Δ, percent change; ρ, Spearman’s rank correlation coefficient; *r*, Pearson’s correlation coefficient.

## DISCUSSION

The novel findings of this study are as follows: *1*) respiratory modulation of MSNA burst occurrence (but not burst area) is diminished in healthy postmenopausal females compared with premenopausal females and younger males, *2*) that respiratory sympathetic modulation is similar among healthy premenopausal females and age-matched males, and *3*) postmenopausal females who had less inspiratory inhibition of MSNA had a higher resting blood pressure.

### Respiratory Modulation of Sympathetic Nerve Activity

In agreement with previous studies (5), the current data demonstrate a reduction in MSNA burst incidence and frequency from the mid-late expiration phase to the inspiration/postinspiration phase of the respiratory cycle in healthy humans. The mechanism underlying respiratory sympathetic modulation is thought to be a coupling between respiratory and sympathetic centers in the brainstem (22). Respiratory sympathetic modulation persists in vagotomized animal models (23, 24) and thus appears to be underpinned by this central coupling mechanism. Lung volumes, via pulmonary stretch afferents, have a regulatory effect on respiratory sympathetic modulation, where sympathetic modulation is enhanced at increased lung volumes (3). Patients with lung transplants, who lack pulmonary stretch afferents, exhibit respiratory sympathetic modulation of a similar magnitude to controls at resting tidal volumes but do not show enhanced modulation at higher tidal volumes (25). As such, the central coupling mechanism appears to drive respiratory sympathetic modulation during normal breathing, and pulmonary stretch becomes an important factor when lung volumes are increased. The physiological significance of respiratory sympathetic modulation is still debated. Other aspects of respiratory hemodynamic modulation, for example, respiratory sinus arrhythmia (26), have been proposed to aid efficient gas exchange. Respiratory sympathetic modulation may have a similar function, perhaps by reducing sympathetic vasoconstriction of the pulmonary vasculature during inspiration to slow pulmonary blood flow. Alternatively, inhibition of sympathetic activity during inspiration may result in reduced afterload, which could act to reduce cardiac workload. These potential functions are yet to be investigated, however. The magnitude of respiratory sympathetic modulation may have consequences for resting levels of MSNA, given that inspiratory pauses in sympathetic outflow would reduce the overall level of MSNA across multiple breaths. In support of this, the data from the current study show that healthy people with less inspiratory inhibition of MSNA have a higher overall resting level of MSNA. This has also been shown in patients with chronic heart failure (4). Therefore, reduced respiratory sympathetic modulation may contribute to age or disease-related increases in MSNA.

### Respiratory Modulation of SNA in Aging Females

The novel findings of the current study show that there is reduced respiratory sympathetic modulation in postmenopausal versus premenopausal females, such that postmenopausal females exhibited a smaller reduction in MSNA during inspiration and a smaller rise in MSNA during expiration compared with the other groups (10 [10]% difference in bursts/100 HB between the respiratory phases in the postmenopausal group vs. 38 [17]% in premenopausal females and 38 [12]% in younger males). This contrasts with previous work in males, where no difference in respiratory sympathetic modulation was observed in older versus younger male participants (5). Along with reduced respiratory sympathetic modulation, there was an overall increase in sympathetic activation in the postmenopausal female group of the current study. It is unclear from the current data whether this overall increase in SNA resulted from the reduction in respiratory sympathetic modulation, or whether an alternative mechanism was driving a general sympathetic activation and the modulatory effect of respiration was masked as a result. However, if altered respiratory modulation was the cause of these changes, reduced respiratory sympathetic modulation could be an important contributor to the age-related rise in SNA in females and therefore the increased risk of hypertension after menopause. Given that Shantsila et al. (5) demonstrated no age-related change to respiratory sympathetic modulation despite an overall increase in SNA in males, there may be alternative mechanisms underlying sympathetic activation in aging males.

The mechanism explaining reduced respiratory sympathetic modulation in older females but not older males (5) is unclear. Given that respiratory modulation of blood pressure (Traube–Hering waves) was also reduced in the current postmenopausal versus premenopausal females, sympathetic modulation via the baroreflex could explain the current data, i.e., smaller perturbations in blood pressure caused reduced modulation of SNA. In support of this, Shantsila et al. (5) found that neither respiratory sympathetic modulation, nor Traube–Hering wave amplitude was different in older versus younger males. However, given that the arterial baroreflex is a closed circuit, it is difficult to determine whether smaller Traube–Hering waves are driving reductions in respiratory-modulated SNA, or whether reductions in respiratory-modulated SNA are producing smaller Traube–Hering waves. Both are plausible explanations for the current observations; however, the present study was not designed to interrogate this. There are potential mechanisms to explain reduced respiratory sympathetic modulation in older females that are not secondary to smaller Traube–Hering waves. For example, the data could be explained by a sex-specific effect of aging on pulmonary stretch afferent activity, via either sensitivity of the pulmonary stretch receptors or changes in lung function. Pulmonary stretch receptor afferent activity may be reduced, which could lead to reduced respiratory sympathetic modulation (3). In support of this, early onset menopause has been linked to increased risk of restrictive lung disease (16), in which a smaller lung volume is achieved for a given negative intrapleural pressure (27). However, there is disagreement about whether age-related decline in lung function differs by sex (11, 28). Although no participant reported respiratory disease in the current study, lung function was not measured. Therefore, the role of lung function in explaining the current results is unclear. The current study did not attempt to separate the effect of menopause from aging. Rather, we aimed to study the effect of female aging, including menopausal transition, on respiratory sympathetic modulation. The effect of acute manipulation of female sex hormone levels on respiratory sympathetic control could be studied using alternative experimental models. However, these studies may be of limited relevance to human health, given that aging and menopause do not occur independently under physiological conditions.

Although postmenopausal females in the current study exhibited a smaller respiratory modulation of MSNA burst occurrence, modulation of burst area was similar in the two female groups. This agrees with Shantsila et al. (5), who showed that older males had a similar respiratory modulation of total MSNA as younger males. Why respiratory modulation of burst occurrence may differ from that of burst area remains unclear. In contrast to burst occurrence, which depends solely on whether sympathetic action potentials are activated (or not) during a given cardiac cycle, burst area additionally depends on the number and amplitude of these action potentials (29). It has been suggested that burst occurrence and burst area are controlled by different areas of nuclei in the brainstem (30) and by different afferent inputs, given the different baroreflex sensitivity of different populations of sympathetic action potentials (31). As such, the mechanisms regulating burst occurrence across the respiratory cycle may differ in postmenopausal females, such that bursts are more likely to occur (even during inspiration). However, mechanisms underlying respiratory control of burst size may still be present in postmenopausal females to the same extent as in premenopausal females.

### Respiratory-Heart Rate and Blood Pressure Coupling

In agreement with previous reports (32, 33), respiratory sinus arrhythmia was reduced in the postmenopausal female group versus the younger group. The physiological significance of respiratory sinus arrhythmia is still debated and as such, the consequences of reduced respiratory modulation, particularly in older females, remain unclear. Some propose that respiratory sinus arrhythmia improves gas exchange by maximizing ventilation-perfusion matching (26), whereas others suggest that a reduction in cardiac work is the primary benefit (34). Indeed, animal models of heart failure suggest that restoration of respiratory-modulated heart rate improves cardiac function and structure (35). However, the importance of reduced respiratory sinus arrhythmia in healthy humans like those in the current study remains unclear, given that they lack apparent cardiovascular disease. The current data also show smaller amplitude Traube–Hering waves in the postmenopausal group compared with the younger groups, in contrast to Shantsila et al. (5), who reported similar amplitude Traube–Hering waves in younger and older males. The smaller Traube–Hering waves in the current study indicate that smaller amplitude oscillations in blood pressure during the respiratory cycle occurred in postmenopausal females. Given that Traube–Hering wave amplitude was similarly correlated with respiratory-modulated MSNA across the participants groups of the current study (Supplemental Fig. S1), the association between Traube–Hering waves and MSNA does not appear to be reduced in postmenopausal females. Therefore, the smaller Traube–Hering wave amplitude in the postmenopausal female group is likely to be due to the smaller degree of respiratory sympathetic modulation also observed in this group.

### Resting Blood Pressure and Respiratory Control of SNA 

Finally, the current study investigated whether the magnitude of respiratory sympathetic modulation was associated with resting hemodynamic variables. Only the postmenopausal female group showed an association between resting blood pressure and magnitude of respiratory sympathetic modulation (Table 3), where postmenopausal females who had less inhibition of SNA during inspiration had higher resting blood pressure. The cause of this association is unclear and requires further validation given that in previous studies similar levels of respiratory sympathetic modulation were observed in patients with hypertension and normotensive controls (36). This study only included three females in the hypertensive group, however, menopausal status was not described. The current correlations between resting blood pressure and respiratory sympathetic modulation in postmenopausal but not premenopausal females may be a reflection of the age-related changes in coupling between SNA and blood pressure. In young males and females, resting blood pressure is uncoupled from resting sympathetic nerve activity, whereas older males and females show a positive relationship (7). Thus, if there was tighter coupling between SNA and blood pressure in the current postmenopausal female group, those with reduced respiratory sympathetic modulation may have a higher level of overall sympathetic activity, and hence greater blood pressure.

### Limitations

In addition to the lack of lung function measurements, the current study has several limitations. MSNA was only recorded during normal, quiet breathing, and as such, conditions of enhanced pulmonary stretch receptor afferent activity (deliberate deep breathing) were not available. Furthermore, other methods of measuring respiration (spirometry) would have allowed for normalization of respiration to vital capacity. Finally, although the data demonstrate reduced respiratory sympathetic modulation in postmenopausal females, there is insufficient evidence to conclude whether this occurs via a smaller inhibition of SNA with inspiration, reduced SNA with expiration, or a combination thereof. As such, it is difficult to determine the brainstem mechanism driving altered respiratory modulation of SNA in postmenopausal females.

## DATA AVAILABILITY

Data will be made available upon reasonable request.

## SUPPLEMENTAL DATA

10.6084/m9.figshare.22673056 Supplemental Fig. S1 and Tables S1–S3: https://doi.org/10.6084/m9.figshare.22673056.

## GRANTS

Z.H.A. was funded by British Heart Foundation Nonclinical PhD Studentship Grant FS/17/38/32935 and University of Bristol British Heart Foundation Accelerator Award AA/18/1/34219. Data collection was supported by funding from National Heart, Lung, and Blood Institute Grant HL083947 (to M.J.J. and N.C.) and American Heart Association Grant 070036Z (to E.C.H.).

## DISCLOSURES

No conflicts of interest, financial or otherwise, are declared by the authors.

## AUTHOR CONTRIBUTIONS

Z.H.A., H.C.B., and E.C.H. conceived and designed research; N.C., T.B.C., M.J.J., and E.C.H. performed experiments; Z.H.A. analyzed data; Z.H.A., H.C.B., M.J.J., A.H.K., A.P.A.S., and E.C.H. interpreted results of experiments; Z.H.A. prepared figures; Z.H.A. drafted manuscript; Z.H.A., H.C.B., N.C., T.B.C., M.J.J., A.H.K., A.K.N., A.P.A.S., and E.C.H. edited and revised manuscript; Z.H.A., H.C.B., N.C., T.B.C., M.J.J., A.H.K., A.K.N., A.P.A.S., and E.C.H. approved final version of manuscript.
